# Supplementary material for: Direct Patlak Reconstruction of [68Ga]Ga-PSMA PET for the Evaluation of Primary Prostate Cancer Prior Total Prostatectomy: Results of a Pilot Study
Source: Int J Mol Sci. 2023 Sep 5;24(18):13677. doi: 10.3390/ijms241813677 (PMC10530818; doi:10.3390/ijms241813677)
Supplement: Supplementary file 1 [file ijms-24-13677-s001.zip › ijms-2489411-supplementary.pdf]

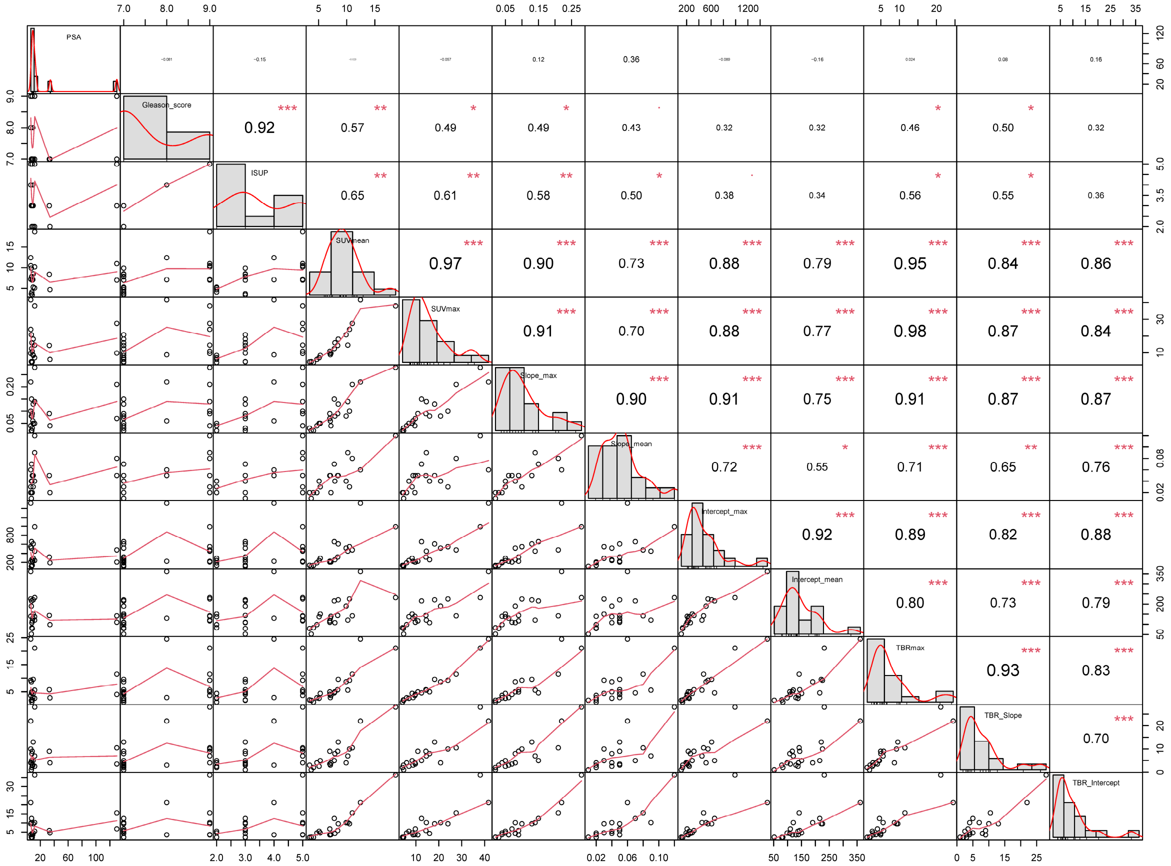

Figure S1: Values of Spearman's correlation coefficients analysis of all extracted static and dynamic imaging parameters in relation to Gleason score and ISUP grading. (*) weak correlations. (**) moderate correlations. (***) strong correlations.
